# Obesity is not a risk factor for either mortality or complications after laparoscopic cholecystectomy for cholecystitis

**DOI:** 10.1038/s41598-021-81963-5

**Published:** 2021-01-27

**Authors:** Yuta Enami, Takeshi Aoki, Kodai Tomioka, Tomoki Hakozaki, Takahito Hirai, Hideki Shibata, Kazuhiko Saito, Yojiro Takano, Junichi Seki, Sonoko Oae, Shoji Shimada, Kenta Nakahara, Yusuke Takehara, Shumpei Mukai, Naruhiko Sawada, Fumio Ishida, Masahiko Murakami, Shin-ei Kudo

**Affiliations:** 1 Digestive Disease Center, Showa University Northern Yokohama Hospital, 35-1 Chigasaki-chuo, Tsuzuki-ku, Yokohama, 224-8503 Japan; 2 Department of Gastrointestinal and General Surgery, Showa University, School of Medicine, 1-5-8, Hatanodai, Shinagawa-ku, Tokyo, 142-8666 Japan

**Keywords:** Cholecystitis, Cholelithiasis

## Abstract

Obesity is a positive predictor of surgical morbidity. There are few reports of laparoscopic cholecystectomy (LC) outcomes in obese patients. This study aimed to clarify this relationship. This retrospective study included patients who underwent LC at Showa University Northern Yokohama Hospital between January 2017 and April 2020. A total of 563 cases were examined and divided into two groups: obese (n = 142) (BMI ≥ 25 kg/m^2^) and non-obese (n = 241) (BMI < 25 kg/m^2^). The non-obese group had more female patients (54%), whereas the obese group had more male patients (59.1%). The obese group was younger (56.6 years). Preoperative laboratory data of liver function were within the normal range. The obese group had a significantly higher white blood cell (WBC) count (6420/μL), although this was within normal range. Operative time was significantly longer in the obese group (*p* = 0.0001). However, blood loss and conversion rate were not significantly different among the groups, neither were surgical outcomes, including postoperative hospital stay and complications. Male sex and previous abdominal surgery were risk factors for conversion, and only advanced age (≥ 79 years) was an independent predictor of postoperative complications as observed in the multivariate analysis. Although the operation time was prolonged in obese patients, operative factors and outcomes were not. Therefore, LC could be safely performed in obese patients with similar efficacy as in non-obese patients.

## Introduction

Obesity has become a global problem. An increasing obese population has become a national affliction not only in the United States^1^ and Europe but also in Asia and Japan^2^. Moreover, in the last 10 years, the prevalence of obesity has increased with age in Japan^3^, and the transition from overweight to obese has become greater in women than in men^4^.

Obesity has been reported as a positive predictor of surgical morbidity. Some previous reports have shown that obesity is a positive predictor of surgical morbidity or poor prognosis, for procedures such as gastrectomy^5^, colectomy^6^, hepatectomy^7–9^, and laparoscopic cholecystectomy (LC)^10,11^. Meanwhile, others have reported that obesity does not increase morbidity or mortality after gastrectomy^12^ and hepatectomy^13^.

LC is the standard operation for benign gallbladder lesions. Once complications occur, damage to the quality of live in patients is very deep. Only a few reports have assessed the impact of obesity-related comorbidities on the operative risk for patients after LC. This study aimed to compare outcomes between obese and non-obese patients after LC.

## Materials and methods

**Figure 1:**
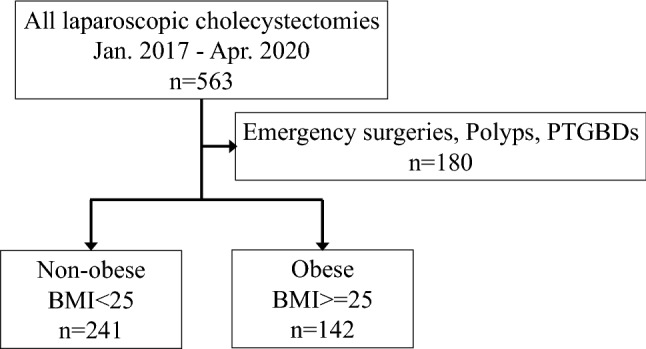
Flow chart of this study. BMI, body mass index; PTGBD, percutaneous transhepatic gallbladder drainage.

A retrospective study was performed on patients who underwent LC at Showa University Northern Yokohama Hospital from January 2017 to April 2020. A total of 563 cases were examined. Data were divided into two groups: the obese group (n = 142) (body mass index [BMI] ≥ 25 kg/m^2^) and non-obese group (n = 241) (BMI < 25 kg/m^2^) (Fig. 1). BMI criteria were defined using the World Health Organization (WHO) classification cutoff value for Asian populations^14^.

Patient characteristics included age, sex, height, body weight, and BMI. Preoperative data included laboratory data, American Society of Anesthetists (ASA) physical status, previous abdominal surgery, and preoperative complications. Operative factors included total operative time and estimated blood loss. Postoperative outcomes, including conversion rate, postoperative complications, and hospital stays, were retrospectively compared between the two groups. For standardization purposes, complications were classified according to the Clavien-Dindo classification system^15,16^. Patients with Clavien-Dindo grade 2 or higher were deemed to have a complication. All LCs were performed for symptomatic gallstone disease with cholecystitis. We excluded emergency surgery, LCs for suspicious malignant diseases, or surgeries with percutaneous transhepatic gallbladder drainage. At our facility, emergency surgeries are usually performed at night, when the number of surgical staff is limited. Emergency surgery cases were excluded because scrub nurses are usually absent, which is likely to affect the operative time.

The indication for LC was benign gallbladder disease in all patients. Obesity has never been considered a contraindication for LC. All LCs were performed using the conventional 4-port technique in the supine position under general anesthesia. A trans-umbilical vertical incision was made with a 12-mm trocar using the open technique in the umbilicus to achieve carbon dioxide insufflation, and the intraabdominal pressure was maintained at 10 mmHg, and a 30° laparoscope was used through a 12-mm trocar. Then, one 5-mm trocar was inserted beneath the xiphoid, and two 5-mm trocars were inserted below the right costal arch. Intraoperative cholangiography was not performed routinely. Multiple surgeons performed the procedures.

Data analysis was performed using JMPPro 14 (SAS Institute Inc., Cary, NC, USA). For comparisons between the groups, a Chi-square test was used for categorical variables, and a Student's *t* test was used for quantitative variables. The results are expressed as the mean ± standard deviation. Multivariate logistic regression was used to identify other factors associated with increased conversion and postoperative complication rates. Probability *p*-values were considered statistically significant at < 0.05 level.

The Committee on Ethics of Showa University Medical School and IRB (Institutional Review Board) reviewed and approved the study protocols (approval code: No. 19H048), and all patients provided written informed consent. All procedures in this study were performed in accordance with the relevant guidelines and regulations.

## Results

The results of patient characteristics are shown in Table 1. The non-obese group had a higher percentage of female patients (54%) whereas the obese group had a higher percentage of male patients (59%). However, there were 7 patients with a BMI > 35, and all of them were female (data not shown). The obese group was comprised of younger participants (56.6 y). There was no significant difference in height between the two groups. Mean BMI was 28.2 (kg/m^2^) in the obese group and 21.7 (kg/m^2^) in the non-obese group.

**Table 1:** Patients characteristics.

	Non-obese (control)	Obese	*p* value
	n = 241	n = 142	
Female	131 (54%)	58 (40.9%)	0.0106
Age (y)	60.2 ± 14.2	56.6 ± 13.0	0.0144
Height (cm)	162.2 ± 8.9	163.8 ± 9.4	0.0836
BMI (kg/m^2^)	21.7 ± 2.1	28.2 ± 3.2	< 0.0001

In the obese group, the range was 25–40.2 kg/m^2^, and in the non-obese group, the range was 14.6–24.9 kg/m^2^.

BMI, body mass index.

All preoperative laboratory data of liver function before surgery were within the normal range, and there was no significant difference between the two groups. However, there was a significant difference in the white blood cell (WBC) count in each group (Table 2). The higher WBC count (6420/μL) was in the obese group, although the WBC count remained in the normal range.

**Table 2:** Preoperative laboratory data.

	Non-obese (control)	Obese	*p* value
	n = 241	n = 142	
WBC (/μL)	5660 ± 1525	6420 ± 1669	< 0.0001
PL (× 10^4^/μL)	23.6 ± 6.6	24.3 ± 6.3	0.3155
CRP (mg/dL)	0.31 ± 0.77	0.52 ± 1.11	0.0536
T-Bil (mg/dL)	0.70 ± 0.30	0.69 ± 0.49	0.7531
AST (IU/L)	26.0 ± 20.1	31.7 ± 51.9	0.1544
ALT (IU/L)	30.8 ± 52.3	39.0 ± 58.6	0.1832
PT (%)	96.0 ± 9.1	97.3 ± 8.1	0.2023

WBC, white blood cell; PL, platelet; CRP, C-reactive protein; T-Bil, total bilirubin; AST, aspartate aminotransferase; ALT, alanine aminotransferase; PT, prothrombin time.

Preoperative factors are presented in Table 3. There were no significant differences in the American Society of Anesthetists classification scores; however, the scores were higher in the obese group. The rate of patients with a previous laparotomy was almost 25%, which was not significantly different between the two groups.

**Table 3:** Preoperative factors.

	Non-obese (Control)	Obese	*p* value
	n = 241	n = 142	
**ASA score**			0.0596
1	108 (44.8%)	51 (35.9%)	
2	130 (53.9%)	85 (59.9%)	
3	3 (1.2%)	6 (4.2%)	
Previous abdominal surgery	61 (25.3%)	36 (25.5%)	0.9619

ASA, American Society of Anesthetists-physical status.

Regarding preoperative complications, diabetes and liver disease were more common in the obese group than in the non-obese group, and there was a significant difference (Table 4). Moreover, the incidence of gastrointestinal cancer was significantly lower in the obese group than in the non-obese group.

**Table 4:** Preoperative complication.

	Non-obese (control)	Obese	*p* value
	n = 241	n = 142	
Diabetes	18 (7.5%)	20 (14.1%)	0.0365
Hyperlipidemia	25 (10.4%)	18 (12.7%)	0.4905
Ischemic heart disease, PCI	5 (2.1%)	6 (4.2%)	0.2235
Arrhythmia	7 (2.9%)	7 (4.9%)	0.3077
Other heart disease	17 (7.1%)	7 (4.9%)	0.4073
Respiratory disease	14 (5.8%)	10 (7.0%)	0.6305
Liver disease	4 (1.7%)	8 (5.6%)	0.0311
Renal disease	6 (2.5%)	4 (2.8%)	0.8462
Gastrointestinal cancer	18 (7.5%)	3 (2.1%)	0.0261
Other malignant disease	10 (4.2%)	7 (4.9%)	0.7203
Ulcer disease	8 (3.3%)	8 (5.6%)	0.2742
Cerebrovascular disease	7 (2.9%)	2 (1.4%)	0.3505
Aortic disease	2 (0.8%)	3 (2.1%)	0.2854
Collagen disease	10 (4.2%)	3 (2.1%)	0.2877
Psychic disease	4 (1.7%)	4 (2.8%)	0.4444
Other disease	26 (10.8%)	21 (14.8%)	0.2491

PCI, percutaneous coronary intervention.

There were significant differences in the operative time, which was longer in the obese group than in the non-obese group (Table 5). However, considering blood loss (Table 5) and conversion rate (Table 6), there were no significant differences between the two groups.

**Table 5:** Operative factors.

	Non-obese (control)	Obese	*p* value
	n = 241	n = 142	
Operative time (min)	84.3 ± 35.6	99.4 ± 39.1	0.0001
Blood loss (mL)	15.0 ± 49.0	15.3 ± 421.8	0.9441

**Table 6:** Operative outcomes.

	Non-obese (control)	Obese	*p* value
	n = 241	n = 142	
Conversion, n	7 (2.9%)	5 (3.5%)	0.7380
Hospital stay (day)	3.4 ± 1.8	3.5 ± 2.4	0.6263
Postoperative complications, n	3 (1.2%)	2 (1.4%)	0.8916

Finally, significant differences in surgical outcomes, including postoperative hospital stay and complications, were not detected in the two groups (Table 6). The incidence of postoperative complications in the two groups was 1.2–1.4%, with no significant difference observed between them (Table 6).

Moreover, male sex and previous abdominal surgery were risk factors for conversion (Table 7), and only advanced age (≥ 79 years) was an independent predictor of postoperative complications as observed in the multivariate analysis (Table 8).

**Table 7:** Multivariate analysis of factors associated with conversions.

Variables	OR	95% CI	*p* value
Age (< 79 vs. ≥ 79 )	0.42	0.09–3.04	0.3115
Sex ( male vs. female )	17.24	3.08–325.33	0.0081
BMI (< 25 vs. ≥ 25 )	0.79	0.23–2.83	0.7026
Diabetes	0.43	0.02–2.53	0.4404
Liver disease	0.00	0.00–2.90	0.9921
Previous abdominal surgery	4.06	1.10–14.21	0.0279

OR, odds ratio; CI, confidence interval.

**Table 8:** Multivariate analysis of factors associated with postoperative complications.

Variables	OR	95% CI	*p* value
Age (< 79 vs. ≥ 79 )	9.31	1.15–61.37	0.0197
Sex ( male vs. female )	0.22	0.01–1.69	0.1979
BMI (< 25 vs. ≥ 25 )	0.82	0.12–6.62	0.8325
Diabetes	0.00	0.00–0.00	0.9912
Liver disease	0.00	0.00–0.00	0.9955
Previous abdominal surgery	0.98	0.12–20.57	0.9835

OR, odds ratio; CI, confidence interval.

## Discussion

Currently, obesity is a global problem. It has become a greater problem not only in Western countries but also in Japan. Our study showed that the higher the BMI, the higher the proportion of women and young people. This is similar to the findings of a previous report^10^ and seems to be a global trend.

According to WHO^14^, the Asian population has a different association between BMI, body fat percentage, and health risk than the European population. Therefore, it can be assumed that the health risk cut-off point for Asians is lower than the existing WHO cut-off point. Some reports used 25 kg/m^2^ as a BMI cut-off point for obesity in the Japanese^5^ and Korean^17^ populations, respectively. Considering that we are an Asian (Japanese) population, we also used 25 kg/m^2^ as a BMI cut-off point for obesity in our study.

The preoperative WBC count was significantly higher with a higher BMI. The exact mechanisms for preoperative elevation of WBC are unexplained, but it has been reported that the incidence of histological liver damage is very high in obese patients^18,19^. This may be a reason why the WBC count tends to increase.

The higher the BMI of the patient, the worse the ASA score (Table 3). It is thought that this is because diabetes and liver disease are common in obesity^18–20^. These preoperative complications were more frequent in obesity, and there was also a significant difference in our results (Table 4). However, it is unlikely that preoperative complications influenced the operative time and postoperative results. Moreover, gastrointestinal cancer was significantly more common in the non-obese group, probably because patients with a history of cancer are less likely to become obese. Especially in postoperative patients with gastric cancer, it is said that there are many cases of weight loss, as it was reported that 67% of patients lost more than 10% of their body weight after surgery^21^. In other words, it seems that the interpretation that the history of cancer is almost unrelated to obesity is correct rather than the interpretation that it is more common in the non-obese group.

According to various reports, patients who had a longer operation time in LC were more likely to have biliary injury^22^, and obese patients had a higher conversion rate^10^ as well as a higher proportion of biliary injury^11^. However, our data showed no significant difference in intra- and postoperative complications, and even in obese patients, postoperative complications were as low as 1.4%. In addition, there were no cases of intraoperative biliary tract injury, and all LCs could be performed safely. As for the surgical factors, the higher the BMI of the patient, the longer the operative time, but the amount of bleeding was not significantly different.

Although it is recommended to confirm the “critical view of safety” (CVS) to safely perform LC^23^, the operative time was significantly longer in obese patients because the amount of fat in the abdominal cavity was large which took a long time to remove and, probably, also to confirm CVS. In addition, when it is difficult to confirm the critical view of safety, the fundus-first technique (dome down, antegrade dissection) may have been adopted more often without being particular about confirmation of CVS, which may have lengthened the operative time^24,25^. We have adopted this method because there are reports that it is safer to complete cholecystectomy by switching to a method such as the fundus-first technique^24,25^. Additionally, it was difficult to dissect the gallbladder from the gallbladder bed. Furthermore, since obese patients have thick subcutaneous fat and a depressed umbilicus, it is very difficult to close the abdominal wall, which could be considered as one of the factors that make the operative time prolonged compared to non-obese patients.

However, there were no significant differences in the length of hospital stay, conversion rate, or postoperative complication rate, except operative time. Moreover, there were no intraoperative complications such as iatrogenic biliary tract injury, which is considered as one of the more serious complications. Furthermore, there were no unfortunate cases of postoperative death (Table 9).

**Table 9:** Postoperative complications.

	Non-obese (control)	Obese
	n = 241	n = 142
Heart dysfunction	0	1
Intestinal injury	1	1
Cerebral bleeding	1	0
Bile leakage	1	0
Mortality	0	0

Chang et al.^26^ reported that only acute cholecystitis was a predictor for conversion and complications in Taiwanese patients. Neylan et al.^27^ analyzed cases of acute cholecystitis and reported that an intended open procedure and conversion were associated with an increased risk of death and serious morbidity. In addition, Franks et al.^28^ reported that male sex and emergent cholecystectomy were independent predictors of increased conversions and complications, and diabetes was a risk factor for conversion, whereas age > 65 years was a risk factor for complications. On the other hand, in our study, scheduled surgeries for cholecystitis were all laparoscopic cholecystectomies which did not include the intended laparotomy, hence it can be considered as a study that eliminates such biases. In addition, the bias of emergency surgeries was eliminated in our study. Furthermore, our study targeted a population of Asians, especially the Japanese. Of note, we found that male sex and a history of abdominal surgery were risk factors for conversion, and only advanced age (≥ 79 years) was an independent predictor of postoperative complications as observed in the multivariate analysis.

The degree of obesity in a patient affected and prolonged the operation time but did not affect the patient's outcome in this study.

The limitations of our study are that the analysis was limited to a single institution and that the study had a retrospective design. Further analysis and more patients are needed to confirm the effect of our study.

In conclusion, although the operation time was prolonged in obese patients, the bleeding volume, conversion rate, complications, and length of stay in the hospital were not prolonged; therefore, LC could also be safely performed in obese patients with similar efficacy as in non-obese patients.
